# Biochemical Characterization of Arylamine *N-*acetyltransferases From *Vibrio vulnificus*

**DOI:** 10.3389/fmicb.2020.595083

**Published:** 2021-01-18

**Authors:** Xinning Liu, Yuanchang Liu, Guangjian Zhao, Yidan Zhang, Lu Liu, Juan Wang, Yifan Wang, Siyu Zhang, Xin Li, Dongliang Guo, Peng Wang, Ximing Xu

**Affiliations:** ^1^Marine Drug Screening and Evaluation Platform (QNLM), School of Medicine and Pharmacy, Ocean University of China, Qingdao, China; ^2^Center for Innovation Marine Drug Screening & Evaluation, Pilot National Laboratory for Marine Science and Technology (Qingdao), Qingdao, China; ^3^Institute of Bioinformatics and Medical Engineering, Jiangsu University of Technology, Changzhou, China; ^4^Quality Control Department, Qilu Children’s Hospital of Shandong University, Jinan, China; ^5^College of Food Science and Engineering, Ocean University of China, Qingdao, China; ^6^School of Life Sciences, Lanzhou University, Lanzhou, China; ^7^School of Information Science and Engineering, Yanshan University, Qinhuangdao, China

**Keywords:** Arylamine *N*-acetyltransferases, *Vibrio vulnificus*, enzyme, acetylation, acetyl coenzyme A

## Abstract

*Vibrio vulnificus* is a zoonotic bacterium that is capable of causing highly lethal diseases in humans; this pathogen is responsible for 95% of all seafood-related deaths in the United States. Arylamine *N*-acetyltransferases (NAT, E.C. 2.3.1.5) is a major family of xenobiotic-metabolizing enzymes that can biotransform aromatic amine chemicals. In this research, to evaluate the effect of NAT on acetyl group transformation in arylamine antibiotics, we first used sequence alignment to study the structure of *V. vulnificus* NAT [(VIBVN)NAT]. The *nat* gene encodes a protein of 260 amino acids, which has an approximate molecular mass of 30 kDa. Then we purified recombinant (VIBVN)NAT and determined the enzyme activity by PNPA and DTNB methods. The DTNB method indicates that this prokaryotic NAT has a particular substrate specificity towards aromatic substrates. However, (VIBVN)NAT lost most of its activity after treatment with high concentrations of urea and H_2_O_2_. In addition, we also explored the stability of the enzyme at different temperatures and pH values. In analyzing the influence of metal ions, the enzyme activity was significantly inhibited by Zn^2+^ and Cu^2+^. The kinetic parameters *K*_*m*_ and *V*_*max*_ were determined using hydralazine, isoniazid, *4*-amino salicylic acid, and *4*-chloro-*3*-methylaniline as substrates, and the *T*_*m*_, *T*_*agg*_ and size distribution of (VIBVN)NAT were observed. In particular, a molecular docking study on the structure of (VIBVN)NAT was conducted to understand its biochemical traits. These results showed that (VIBVN)NAT could acetylate various aromatic amine substrates and contribute to arylamine antibiotic resistance in *V. vulnificus*.

## Introduction

*Vibrio vulnificus* (*V. vulnificus*) is a ubiquitous gram-negative aquatic bacterium that belongs to the Vibrionaceae family. *V. vulnificus* has been recognized as one of the most diverse and dangerous pathogens worldwide (Centers for Disease Control Prevention [CDC], 2013; Oliver, 2015). Approximately 95% of all seafood-related deaths in the United States are caused by this highly lethal pathogen, with a mortality rate of approximately 50% in immunocompromised and high-risk populations (Liu et al., 2006; Jones and Oliver, 2009). *V. vulnificus* can generally invade human hosts through two routes: oral consumption and wound infection. Oral consumption of seafood such as, primarily, raw oysters or raw molluscan shellfish, can cause severe gastroenteritis or septicemia infection (Kim et al., 2015). Wound infection is generally acquired by exposure to contaminated seawater or seafood products, resulting in necrotizing fasciitis (Huang et al., 2016). It has been reported that *V. vulnificus* preferentially grows in warm (above 20°C), low-salinity (<25 ppt NaCl) seawater (Baker-Austin et al., 2010). Due to global warming, the incidence of this infection has increased dramatically worldwide because of the spreading geographical distribution of *V. vulnificus* infection, and the disease was found even in some previously unaffected regions (Vezzulli et al., 2013). *V. vulnificus* infections are characterized as a short time-span infection. Without antibiotic treatment, the mortality rate of infection will dramatically increase from 33 to 100% in less than 48 h (Klontz et al., 1988). Thus, the need to find an accurate and rapid treatment of this bacterium in clinical settings is paramount. In previous investigations, *V. vulnificus* was usually susceptible to a great variety of antibiotics such as tetracyclines, aminoglycosides, third-generation cephalosporins, chloramphenicol, and newer fluoroquinolones (Morris and Tenney, 1985; Tang et al., 2002; Baker-Austin et al., 2009). However, more recently, it has been suggested that *V. vulnificus* has emerging resistance to various antibiotics (Cabello, 2006; Shaw et al., 2014; Elmahdi et al., 2016; Serratore et al., 2017). Hence, understanding the resistance mechanism of *V. vulnificus* is urgently needed.

Arylamine *N*-acetyltransferases (NATs, E.C. 2.3.1.5) are cytosolic phase II xenobiotic-metabolizing enzymes (XMEs) that are widely distributed in prokaryotes and eukaryotes. They catalyze acetyl group transfer from acetyl-CoA (AcCoA) to arylamines, arylhydrazines, and *N*-hydroxyarylamine and play an essential role in the detoxification and/or bioactivation of numerous drugs and carcinogens (Riddle and Jencks, 1971; Sim et al., 2008b). NAT enzymes have been identified and characterized in a range of mammals, bacteria, fungi, and other major taxonomic groups (Glenn et al., 2010; Martins et al., 2010). In humans, there are two polymorphic NAT isoforms (NAT1 and NAT2) that have distinct expression patterns and functional implications (Blum et al., 1990). (HUMAN)NAT1 is ubiquitously expressed and specific for *p*-aminosalicylate, *p*-aminobenzoic acid, folate catabolite, and *p*-aminobenzoylglutamate (Rodrigues-Lima et al., 2010). (HUMAN)NAT2 is expressed in a restricted range of tissues and responsible for the inactivation of the front-line antitubercular drug isoniazid (INH) (Evans et al., 1960). In 2,000, the structure of NAT from *Salmonella typhimurium* was determined as the first NAT crystal structure. The NAT structure revealed a Cys-His-Asp catalytic triad, which is also present in transglutaminase, deubiquitinase, and cysteine protease (Sinclair et al., 2000). In bacteria, NAT-dependent *N*-acetylation could be employed as a defense mechanism against environmental toxins by acetylating and inactivating different arylamines. The transformation of the *nat* gene from *Mycobacterium tuberculosis* (MYCTU) into *Escherichia coli* will increase the resistance to INH 3-fold in *E. coli* (Payton et al., 1999). Subsequent studies reported that bacteria with the deleted *nat* gene, such as *Mycobacterium smegmatis* (MYCSM) and *Mycobacterium bovis bacille Calmette-Guérin* (*M. bovis BCG*), were more sensitive to INH (Payton et al., 2001; Bhakta et al., 2004). Mycobacterial NAT enzymes are therefore promising therapeutic targets for the development of antimycobacterial compounds. Here, we purified and characterized the NAT enzyme from *V. vulnificus*. We used biochemical approaches to describe the enzymatic properties, substrate specificity, physicochemical properties, and kinetic parameters of the enzyme and used sequence alignments and molecular modeling to analyze the similarity of (VIBVN)NAT with other species, the structure of the enzyme in complex with CoA and AcCoA, and the substrate binding mode.

## Materials and Methods

### Bacterial Strains and Biological Reagents

*Escherichia coli* BL21(DE3) (F^–^ *omp*T *hsd*SB (r_*B*_^–^m_*B*_^–^) *gal dcm*) (Shanghai Weidi Biotechnology Co., Ltd., Shanghai, China) and *E. coli* DH5α (dlacZ Delta M15 Delta (lacZYA-argF) U169 recA1 endA1 hsdR17(rK-mK+) supE44 thi-1 gyrA96 relA1) were used for protein expression and cloning, respectively (Studier and Moffatt, 1986; Taylor et al., 1993). A prepacked desalting column and Ni Sepharose column were purchased from Smart Lifesciences (Changzhou China). The pET28a+ vector from Personalbio (Shanghai, China) was used to express recombinant proteins. Isopropylthio-β-*D*-galactoside (IPTG) and kanamycin were obtained from Solarbio Science & Technology Co., Ltd. (Beijing, China). Ethylenediaminetetraacetic acid (EDTA), 1,4-dithiothreitol (DTT), 5,5′dithiobis-(*2*-nitrobenzoic acid) (DTNB), acetyl coenzyme A trilithium salt (AcCoA), and enzyme substrates including *2*-aminofluorene (2-AF), hydralazine (HDZ), *5*-aminosalicylate (5-AS), *4*-amino salicylic (4-AS), sulfamethoxazole (SMX), INH, *4*-chloro-*3*-methylaniline (4-C3ME) and *4*-aminobenzoic acid (pABA) were purchased from Aladdin (Shanghai, China).

### Sequence Analysis

The amino acid sequence of (VIBVN)NAT was obtained from the UniProt database (A0A4V3BB88). Multiple amino acid sequences of NAT from different species were aligned using the MUSCLE program, and the result was displayed by ESPript 3.0^1^. The sequence analysis involved 12 amino acid sequences from *Bacillus cereus* (BACCR), *Bacillus anthracis* (BACAN), *Homo sapiens* (HUMAN), *Pseudomonas aeruginosa* (PSEAE), *S. typhimurium* (SALTY), *Rhizobium loti* (RHILO), MYCSM, *Mycobacterium abscessus* (MYCA9), *Nocardia farcinica* (NOCFA), *Mycobacterium marinum* (MYCMR), and *M. tuberculosis* (MYCTU).

### Cloning, Expression, Purification, and Molecular Weight Determination of *V. vulnificus* Arylamine *N*-acetyltransferase

The target gene was synthesized and subcloned into the NotI and BamHI restriction sites in the pET-28a(+) expression vector and transformed into *E. coli* DH5α cells by Personalbio (Shanghai, China). The recombinant plasmid was then transformed to obtain the (VIBVN)NAT protein in *E. coli* BL21(DE3) cells. After that, a single colony of *E. coli* BL21(DE3) was inoculated into LB medium containing kanamycin (50 μg/mL) and incubated on a rotary shaker at 37°C until the optical density at 600 nm (OD600) reached approximately 0.8. Then, IPTG was added to a final concentration of 200 μM to induce enzyme expression, and the bacterial culture was further grown for 18 h at 16°C. The resulting cells were harvested and sonicated in a buffer solution containing 25 mM Tris–HCl (pH 7.5), 150 mM sodium chloride, 20 mM imidazole, 0.2 mg/mL lysozyme, 1 mM MgCl_2_, 1 mM PMSF and 0.05% Triton X-100. Cellular debris was removed by centrifugation at 12000 rpm for 45 min at 4°C. The supernatants were loaded onto a Ni Sepharose column followed by extensive washing with washing buffer [25 mM Tris–HCl (pH 7.5), 150 mM NaCl, and 20 mM imidazole]. The (VIBVN)NAT proteins were then gradient eluted with Tris–HCl buffer containing 40 to 200 mM imidazole and desalted using a prepacked column with 25 mM Tris–HCl (pH 7.5) and 150 mM NaCl. The final protein was stored at −80°C without further modifications. The purity of (VIBVN)NAT was confirmed by sodium dodecyl sulfate polyacrylamide gel electrophoresis (SDS-PAGE). SDS-PAGE was performed in 12% polyacrylamide gels according to the method of Laemmli (1970). The separated proteins were stained with Coomassie Brilliant Blue R-250, and their molecular weights were determined by comparing their mobility with that of the standard prestained markers of known molecular weights (range of 15–180 kDa).

### Gel Filtration Chromatography

The purified (VIBVN)NAT was subjected to gel filtration chromatography analysis, and chromatographic separations were performed using Superdex 75 with a flow rate of 0.5 ml/min. The sample was prepared at a concentration of 5 mg/mL in PBS buffer. Approximately 100 μl of sample was injected onto the column. The detector (Qite, China) was adjusted to 280 nm, and the running time was 50 min. The retention time for each peak was noted.

### NAT Activity Assay

To test the enzyme activity of (VIBVN)NAT, we analyzed its catalytic activity by the PNPA and DTNB methods. PNPA method uses PNPA as the acetyl donor. The deacetylated form, *p*-nitrophenol (PNP), absorbs light at 405 nm. For the assay, 80 μL of NAT enzyme (5–40 μg/mL, final concentration) was mixed with 10 μL of arylamine substrate (500 μM, final concentration), and the reaction was initiated by adding 10 μL of PNPA (2 mM, final concentration). All the reagents were diluted in 25 mM Tris–HCl buffer (pH 7.5). The reaction rate was determined by continuously monitoring the absorbance at 405 nm using a plate reader (Molecular Devices). The data were corrected by subtracting the nonspecific hydrolysis of PNPA in the absence of NAT. The measurement of AcCoA-dependent acetylation was carried out using *5*,*5*′-dithiobis-(*2*-nitrobenzoic acid) (DTNB or Ellman’s reagent) as described previously (Brooke et al., 2003). Similar to the PNPA method, 80 μL of NAT enzyme (5–40 μg/mL, final concentration) was mixed with 10 μL of arylamine substrate (500 μM, final concentration) at room temperature, and the reaction was started by adding 10 μL of AcCoA (500 μM, final concentration). After a given time, the reaction was stopped by adding 50 μL of cold DTNB (2.0 mg/mL, final concentration) in 6 M guanidinium chloride (GdmCl). Assays were carried out in 25 mM Tris–HCl buffer (pH 7.5), and the rate was determined by the linear change in absorbance at 405 nm.

### Effect of Substrate Specificity and Concentration on Enzyme Activity

To determine the substrate specificity of (VIBVN)NAT, different substrates were added to the reaction mixture at a final concentration of 500 μM. These included 2-AF, HDZ, 5-AS, 4-AS, SMX, INH, 4-C3ME, and pABA. To determine the effect of different substrate concentrations on enzyme activity, the substrates 4-AS, INH, 4-C3ME, and HDZ (final concentrations of 50–800 μM) were examined by the DTNB method to study the catalytic activity of the purified enzyme.

### Effect of Urea and Hydrogen Peroxide on Enzyme Activity

To determine the effects of urea or hydrogen peroxide (H_2_O_2_) on the activity of (VIBVN)NAT, the enzyme was first incubated with urea or H_2_O_2_ in a series of concentrations. The mixture was then diluted 40 times to determine the remaining enzyme activity using the DTNB method with AcCoA as the acetyl acceptor. The final concentration of urea or H_2_O_2_ ranged from 100 mM to 6 M or 25 to 1,000 μM, respectively. In the reactivation experiments, (VIBVN)NAT was first oxidized by H_2_O_2_ and incubated with DTT for 10 min. The mixture was then diluted 40 times to determine the remaining enzyme activity using the DTNB method. The final concentration of H_2_O_2_ or DTT was 1 mM. A control test was conducted in parallel in the absence of the reagent.

### Effect of Metal Ions on Enzyme Activity

To determine the effect of different metal ions on the activity of (VIBVN)NAT, the enzyme was first incubated with each of the listed metal ions (MgSO_4_, MnSO_4_, KCl, NaCl, ZnSO_4_, CaCl_2_, and CuSO_4_). The mixture was then diluted 40 times to determine the remaining enzyme activity using the DTNB method. The final concentration of metal ions was 1 mM. In reactivation experiments, (VIBVN)NAT was first treated with metal ions and incubated with EDTA for 10 min. The mixture was then diluted 40 times to determine the remaining enzyme activity by the DTNB method. The final concentrations of metal ions and EDTA were 1 mM and 0.1–1 mM, respectively. A control test was conducted in parallel in the absence of the reagent.

### Effect of Temperature and pH Value on Enzyme Activity

The effect of temperature on the activity of (VIBVN)NAT was evaluated by the DTNB method. The experiments were performed under different temperatures ranging from 4 to 85°C for 30 min. A control test was conducted at room temperature (25°C). The optimum pH of the purified enzyme was studied at different pH values ranging from 3–11.5 using Britton-Robinson (BR) buffer, which consists of a mixture of 0.04 M H_3_BO_3_, H_3_PO_4_, and CH_3_COOH, and the pH was adjusted with 0.2 M NaOH to pH 3, 5.5, 7.5, 8.5, 10, or 11.5. A control test was conducted at pH 7.5.

### Kinetic Constants of *V. vulnificus* Arylamine *N*-acetyltransferase

The kinetic constants of (VIBVN)NAT were investigated using 4-AS, INH, 4-C3ME, and HDZ as substrates. The Michaelis–Menten constant (*K*_*m*_) and maximal velocity (*V*_*max*_) were determined for the enzyme using the Michaelis–Menten equation. The catalytic rate constant (*k*_*cat*_) was calculated based on *V*_*max*_’s ratio to the total concentration of the enzyme, using 30 kDa as the molecular weight value, and the catalytic efficiency was determined based on the *k*_*cat*_/*K*_*m*_ ratio.

### *T*_*m*_ and *T*_*agg*_ Characterization of *V. vulnificus* Arylamine *N*-acetyltransferase

The melting and aggregation temperatures of (VIBVN)NAT were evaluated with an all-in-one UNcle stability platform (Unchained Labs, CA, United States), allowing simultaneous analysis of the same low-volume sample in high-throughput mode. Nine microliters of each sample were pipetted into an Uncle UNi, quartz capillary array, and differential scanning fluorimetry (DSF) was used to monitor the change in the protein structure upon increasing the temperature from 25 to 95°C at a 0.3°C/min scan rate. The intrinsic fluorescence and light scattering outputs were acquired at an excitation wavelength of 266 nm to record and determine the melting temperature (the midpoint of the unfolding event, *T*_*m*_). At the same time, static light scattering (SLS) at 266 nm was used as an indicator for colloidal stability to determine the aggregation onset temperature (*T*_*agg*_). The size distribution of the same set of samples was simultaneously determined by a DLS module at 660 nm before and after the heating program. The samples were prepared in PBS at three different concentrations (4.8, 1, and 0.04 mg/mL); measurements were made in triplicate and averaged, and standard errors were calculated by UNit analysis software.

### Molecular Modeling

The structure model of (VIBVN)NAT was obtained by homologous modeling using SWISS-MODEL (Waterhouse et al., 2018). The arylamine *N*-acetyltransferase structure model of BACAN (PDB ID: 3LNB, Chain A) was used as a template. The modeled structure of (VIBVN)NAT was aligned to the (MYCMR)NAT1 (PDB ID: 2VFC) structure with CoA binding. Then, the conformation of CoA in (VIBVN)NAT was regenerated by AutoDock local searching (Morris et al., 2009) and minimized using “ligand or protein_near_ligand” in Schrödinger (Jacobson et al., 2004). Finally, residues around CoA and CoA itself are minimized with the OPLS3e force field. Based on the binding model of (VIBVN)NAT and CoA, the structure of CoA was modified to AcCoA, and the binding model of AcCoA with (VIBVN)NAT was obtained in the same way.

We also explored the binding model of four aromatic amine substrates. The cysteine in the active pocket was transformed to acetylated cysteine, and all atoms of the structure model of (VIBVN)NAT were minimized. The structures of aromatic amine substrates were built with Maestro and optimized using the OPLS3e’ force field. The acetylated protein model was docked with optimized aromatic amine substrates by the Glide docking SP mode (Friesner et al., 2006).

### Statistical Analysis

Graph production, data distribution, and statistical analyses were performed using QtiPlot. Analysis of *t*-tests was used to investigate significant differences between the indicated groups. The data are presented as the mean ± SD of three independent experiments. ^∗^*p* < 0.05, ^∗∗^*p* < 0.01, *p* < 0.05 was considered statistically significant.

## Results

### Sequence Analysis

**FIGURE 1:**
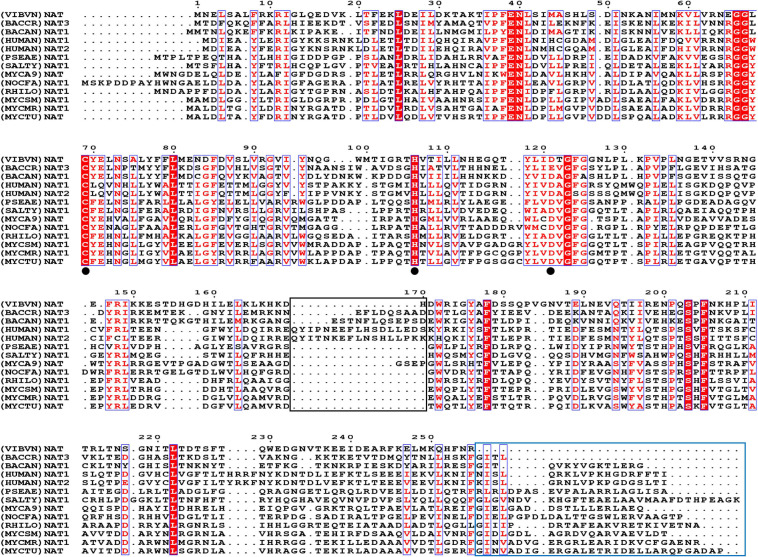
The amino acid sequence alignment results. Comparison of (VIBVN)NAT with 10 bacterial NAT proteins and two human NAT proteins, *Bacillus cereus* (BACCR), *Bacillus anthracis* (BACAN), *Homo sapiens* (HUMAN), *Pseudomonas aeruginosa* (PSEAE), *Salmonella typhimurium* (SALTY), *Rhizobium loti* (RHILO), *Mycobacterium smegmatis* (MYCSM), *Mycobacterium abscessus* (MYCA9), *Nocardia farcinica* (NOCFA), *Mycobacterium marinum* (MYCMR), and *Mycobacterium tuberculosis* (MYCTU). Fully conserved residues are highlighted in a red background, and semi conserved residues are highlighted in red font. The black frame indicates the “mammalian/eukaryotic insertion loop.” The blue frame indicates the lack of the C-terminal region. The catalytic triad is indicated by a black dot.

In this research, we first analyzed the sequence features of (VIBVN)NAT. Multiple sequence alignments of (VIBVN)NAT with sequences of 11 other NAT species were performed. A cysteine protease-like catalytic triad (Cys-His-Asp) is essential for acetyltransferase activity in all NAT homologs (Wang et al., 2004; Sandy et al., 2005a; Wu et al., 2007). Here, we found that (VIBVN)NAT has a typical Cys-His-Asp catalytic triad, which may participate in arylamine acetylation. The catalytic triad is indicated by a black dot in Figure 1. (BACCR)NAT and (BACAN)NAT are closely related to (VIBVN)NAT, with 42 and 41% sequence identity, respectively. We further used the (BACAN)NAT as a template for homology modeling. The C-terminal undecapeptide in NAT is essential for modulating hydrolysis of AcCoA and plays a vital role in substrate and AcCoA binding (Mushtaq et al., 2002). Figure 1 shows that the C-terminal region of (VIBVN)NAT is approximately 20 amino acids shorter than that of other NATs, which may impact the catalytic ability of (VIBVN)NAT. Both eukaryotic and prokaryotic NAT enzymes have a “mammalian/eukaryotic insertion loop.” The role of this loop is not yet fully understood; it may contribute to the binding mode of AcCoA with NAT and maintain the structural integrity of the protein (Wu et al., 2007; Pluvinage et al., 2011). In this research, the amino acid sequence of (VIBVN)NAT was devoid of a “mammalian/eukaryotic insertion loop” (part of the black frame in Figure 1), which suggested that the binding mode of AcCoA with (VIBVN)NAT may exist in a stretched pattern.

### Cloning, Expression, Purification, and Molecular Weight Determination of *V. vulnificus* Arylamine *N*-acetyltransferase

**FIGURE 2:**
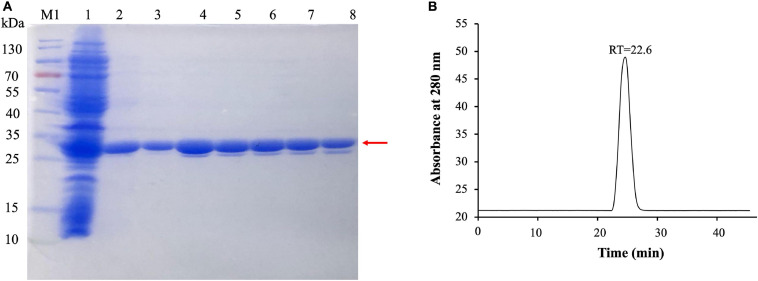
**(A)** SDS-PAGE of the expression and purification of (VIBVN)NAT in *E. coli* BL21(DE3) cells. Detailed legend: Lane M1: Protein marker. Lane 1: Supernatant after cell lysate centrifugation (loading). Lane 2: Flowthrough with 50 mM Tris–HCl, 150 mM NaCl, and 20 mM imidazole (pH 8.0). Lanes 3–5: Elution with 50 mM Tris–HCl, 150 mM NaCl, and 40 mM imidazole (pH 8.0). Lane 6–8: Elution with 50 mM Tris–HCl, 150 mM NaCl, and 200 mM imidazole (pH 8.0). Molecular weight determination by using SDS-PAGE. **(B)** Gel filtration chromatography analysis of (VIBVN)NAT. The enzyme was prepared at a concentration of 5 mg/mL in PBS buffer. Approximately 100 μl of the sample was injected onto the column. The relative amount of the main form of the enzyme (retention time (RT) = 22.6 min) was present in the sample.

To evaluate the activity of (VIBVN)NAT, the enzyme was expressed and purified by Ni-affinity chromatography. The expression of recombinant His-tagged (VIBVN)NAT was analyzed by SDS-PAGE and Western blotting (Figure 2A). Most protein contaminants were removed after one-step affinity chromatography eluted with a linear gradient of imidazole. The purified (VIBVN)NAT protein migrated as a single band on an SDS-PAGE gel (Figure 2A line 3–8). The molecular weight of the purified enzyme was determined by SDS-PAGE according to the method of Laemmli (1970). The purified (VIBVN)NAT protein resulted in a single distinctive band observed with an apparent molecular weight of 30 kDa compared with that of the standard molecular weight markers. This result is consistent with the predicted molecular weight and previous reports on NATs in other kinds of bacteria (Payton et al., 1999; Cocaign et al., 2014). Western blot analysis of (VIBVN)NAT probed with an anti-His tag monoclonal antibody revealed anti-His antibody reactive bands (data not shown). The purity of the final product was confirmed by gel filtration chromatography, as shown in Figure 2B. The chromatogram of the purified (VIBVN)NAT protein displayed a single peak at a retention time of 22.6 min, suggesting that (VIBVN)NAT functions as a stable monomer.

### NAT Activity Assay

**FIGURE 3:**
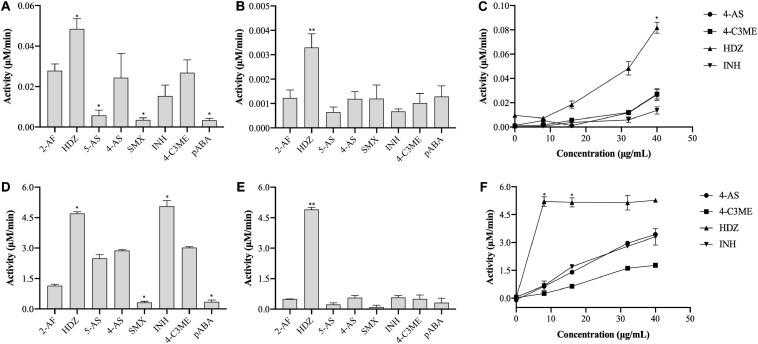
Enzyme activity of (VIBVN)NAT. **(A–C)** The activity of NAT was detected by the PNPA method at **(A)** 40 μg/mL and **(B)** 5 μg/mL. **(C)** A total of 8–40 μg/mL of the enzyme was mixed with 2 mM PNPA and 500 μM acetyl acceptors in 25 mM Tris–HCl buffer (pH 7.5). **(D–F)** The activity of NAT was detected by the DTNB method at **(D)** 40 μg/mL and **(E)** 5 μg/mL. **(F)** A total of 8–40 μg/mL of the enzyme was mixed with 500 μM AcCoA and 500 μM acetyl acceptors in 25 mM Tris–HCl buffer (pH 7.5). The data are presented as the mean ± SD of three independent experiments. Error bars indicate SD values. The results are presented as percent control (4-AS) of the activity of NAT. *p < 0.05, **p < 0.01 when compared to the control group.

In this study, the activity of (VIBVN)NAT was evaluated by the PNPA and DTNB methods with different acetyl donors and acceptors. Through the PNPA assay, we found that (VIBVN)NAT displays weak activity (<0.04 μM/min) for 5-AS, SMX, 4-AS, and pABA at two enzyme concentrations (5 and 40 μg/mL). However, the activity of (VIBVN)NAT was three times higher when HDZ was used as a substrate at a high enzyme concentration ([(VIBVN)NAT] = 40 μg/mL, *p* < 0.01) (Figures 3A,B). Similar data were obtained from the dose-dependent assay with HDZ, 4-AS, INH, and 4-C3ME (8–40 μg/mL) (Figure 3C). A much higher activity profile for the substrates was observed when AcCoA was used as an acetyl donor in the DTNB method (Figures 3D–F), which was approximately 100 times higher than that of the PNPA method. The enzyme was found to acetylate a broad range of substrates such as HDZ, INH, 4-AS, and 4-C3ME. The enzyme acetylation of SMX and pABA was significantly weaker than that of 4-AS (*p* < 0.05). The enzyme activity was considerably higher when HDZ and INH were used as substrates than when 4-AS was used (*p* < 0.05). The activity profile of these substrates observed by the PNPA or DTNB assay suggests that AcCoA is a better acetyl donor than PNPA.

### Effect of Urea and H_2_O_2_ on Enzyme Activity

**FIGURE 4:**
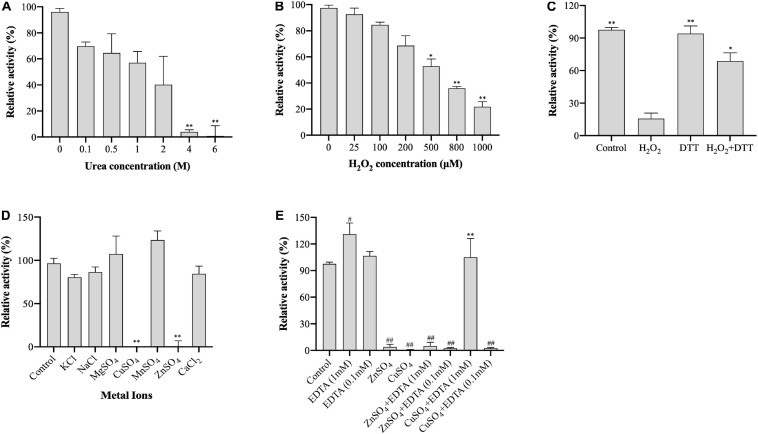
Effect of urea, H_2_O_2_, and metal ions on the activity of (VIBVN)NAT. The activity of NAT was detected by the DTNB method. **(A)** Urea at various concentrations was mixed with 25 μg/mL (final concentration) of the enzyme, 500 μM AcCoA and 500 μM acetyl acceptors in 25 mM Tris–HCl buffer (pH 7.5) at room temperature. The enzyme activity obtained without urea was defined as 100%. The data are presented as the mean ± SD of three independent experiments. Error bars indicate SD values. The results are presented as percent control (urea 0 μM) of the activity of NAT. **p < 0.01 when compared to the control group. **(B)** H_2_O_2_ at various concentrations was mixed with 25 μg/mL (final concentration) of the enzyme, 500 μM AcCoA and 500 μM acetyl acceptor 25 mM Tris–HCl buffer (pH 7.5) at room temperature. The enzyme activity obtained without H_2_O_2_ was defined as 100%. The data are presented as the mean ± SD of three independent experiments. Error bars indicate SD values. The results are presented as percent control (H_2_O_2_ 0 μM) of the activity of NAT. *p < 0.05, **p < 0.01 when compared to the control group. **(C)** H_2_O_2_ at 1 mM (final concentration) was mixed with 25 μg/mL (final concentration) of the enzyme. The oxidized enzyme was then incubated with 1 mM DTT for 10 min. The enzyme activity obtained without H_2_O_2_ and DTT was defined as 100%. The data are presented as the mean ± SD of three independent experiments. Error bars indicate SD values. The results are presented as percent control (H_2_O_2_ 0 μM, DTT 0 μM) of the activity of NAT. *p < 0.05, **p < 0.01 when compared to the H_2_O_2_-inactivated (VIBVN)NAT. **(D)** Different kinds of metal ions were mixed with 25 μg/mL (final concentration) of the enzyme, 500 μM AcCoA and 500 μM acetyl acceptor 25 mM Tris–HCl buffer (pH 7.5) at room temperature. The enzyme activity obtained without metal ions was defined as 100%. The data are presented as the mean ± SD of three independent experiments. Error bars indicate SD values. The results are presented as percent control (no metal ion) of the activity of NAT. **p < 0.01 when compared to the control group. **(E)** ZnSO_4_ and CuSO_4_ were mixed with 25 μg/mL (final concentration) of the enzyme, and then the enzyme was incubated with EDTA at 0.1 or 1 mM for 10 min. The enzyme activity obtained without metal ions and EDTA was defined as 100%. The data are presented as the mean ± SD of three independent experiments. Error bars indicate SD values. The results are presented as percent control (no metal ion, EDTA = 0 μM) of the activity of NAT. **p < 0.01 when compared to the CuSO_4_-inactivated (VIBVN)NAT. ^##^*p* < 0.01, ^#^*p* < 0.05 when compared to the control group.

Urea denaturalizes proteins by destabilizing the internal, noncovalent bonds between atoms. To evaluate the stability of (VIBVN)NAT to urea, the enzyme activity was assessed using the DTNB method under treatment with different urea concentrations. As shown in Figure 4A, the activity of (VIBVN)NAT decreased with increasing urea concentrations, the IC50 value was approximately 2 M, and the enzyme lost most of its activity after treatment with 4 M urea (*p* < 0.01). H_2_O_2_ causes oxidative damage to proteins by producing free radicals. A previous study showed that cysteine residues in the catalytic center of NAT are readily oxidized to cysteine sulfonic acid or disulfide by H_2_O_2_ (Atmane et al., 2003). In this research, sequence analysis showed that (VIBVN)NAT also has a catalytic residue, Cys69. In the analysis of enzyme stability under oxidative stress, the results showed that the activity of (VIBVN)NAT was inhibited by H_2_O_2_ in a dose-dependent manner with an IC50 of 550 μM (Figure 4B). T he enzyme activity decreased remarkably under the treatment of 800 μM H_2_O_2_ (*p* < 0.01). Previous studies showed that the H_2_O_2_-dependent inactivation of (HUMAN)NAT1 could be reversed by thiol-reducing agents such as DTT or GSH (Atmane et al., 2003; Dupret et al., 2005). To investigate whether the inhibition of (VIBVN)NAT by H_2_O_2_ could be reversed by reducing agents, DTT (1 mM final concentration) was added to the inhibition mixture (Figure 4C). The results were consistent with those from previous studies; the H_2_O_2_-dependent inactivation of (VIBVN)NAT was reversed by DTT. Overall, our results showed that both urea and oxidative stress could inactivate (VIBVN)NAT. However, at the same time, the enzyme could also tolerate higher concentrations of urea and H_2_O_2_, indicating that the enzyme has high stability.

### Effect of Metal Ions on Enzyme Activity

Metal ions play a crucial role in maintaining the active configuration of enzymes at elevated temperatures and affecting protein activity by complexing with essential amino acids in proteins (Kochanczyk et al., 2015; Jankiewicz et al., 2020). The effects of different metal ions (1 mM final concentration) on enzyme activity were assessed and are summarized in Figure 4D. The residual activity of the enzyme was significantly varied in the presence of different metal ions. Among all metal ions, the enzyme remained active under the treatment of Mn^2+^ (*p* > 0.05). The activity of (VIBVN)NAT was inhibited in the presence of Zn^2+^ and Cu^2+^ (*p* < 0.01). This result is consistent with an earlier report that papain, which shares a similar Cys-His-Asp catalytic triad with NATs, is inactivated in the presence of Zn^2+^ and Cu^2+^ (Sluyterman, 1967). We further investigated the effect of EDTA on metal ion-dependent inactivation of the enzyme (Figure 4E). The results showed that the enzyme activity was not influenced by 0.1 mM EDTA but slightly increased under 1 mM EDTA (*p* < 0.05). At a concentration of 0.1 mM EDTA, both the Zn^2+^ and Cu^2+^-dependent inactivation of (VIBVN)NAT were completely irreversible. In contrast, EDTA (1 mM final concentration) was able to fully recover the activity of (VIBVN)NAT, which was inactivated by Cu^2+^ (*p* < 0.01), while the inactivation caused by Zn^2+^ was unable to be recovered. Hence, the inhibition of enzyme activity caused by Zn^2+^ was stronger than that caused by the other metal ions.

### Effect of Temperature and pH on Enzyme Activity

**FIGURE 5:**
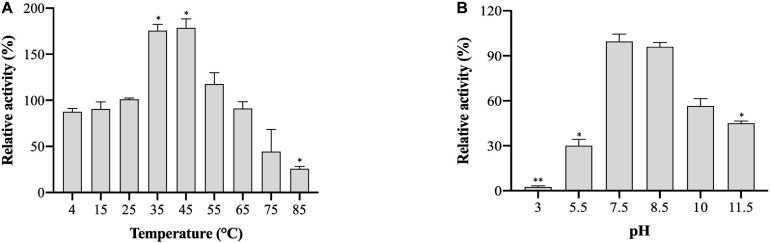
Effect of temperature and pH on the activity of (VIBVN)NAT. The activity of NAT was detected by the DTNB method. **(A)** A total of 25 μg/mL of the enzyme was mixed with 500 μM AcCoA and 500 μM acetyl acceptors in PBS buffer (pH 7.5) at different temperatures. The optimal temperature was determined by measuring enzyme activity at various temperatures under standard assay conditions. The enzyme activity obtained at room temperature (25°C) was defined as 100%. The data are presented as the mean ± SD of three independent experiments. Error bars indicate SD values. The results are presented as percent control (25°C) of the activity of NAT. *p < 0.05 when compared to the control group. **(B)** A total of 25 μg/mL of the enzyme was mixed with 500 μM AcCoA and 500 μM acetyl acceptors at different pH values at room temperature. The optimal pH was measured at room temperature in a BR buffer with distinct pH values under standard assay conditions. The enzyme activity obtained at pH 7.5 was defined as 100%. The data are presented as the mean ± SD of three independent experiments. Error bars indicate SD values. The results are presented as the percent control (pH 7.5) of the activity of NAT. *p < 0.05, **p < 0.01 when compared to the control group.

The effects of temperature and pH on (VIBVN)NAT enzyme activity were studied by the DTNB method. A gradual increase in enzyme activity was noted in a range of temperatures between 4 and 45°C; (VIBVN)NAT showed a relatively high activity at the temperatures of 35 and 45°C compared to that at room temperature (*p* < 0.05). The enzyme rapidly reduced its activity at a relatively high temperature and lost most of its activity at 85°C (*p* < 0.05) with 4-AS as the substrate (Figure 5A). These results showed that the enzyme was more stable at lower temperatures than at higher temperatures. While it has been previously reported that (MYCTU)NAT also has a high heat stability, the enzyme begins to lose activity after incubation at temperatures higher than 60°C (Lack et al., 2009). However, lower heat stability was found in (MYCMR)NAT and (MYCSM)NAT (Kawamura et al., 2003). Analysis of the stability of (VIBVN)NAT at different pH values is illustrated in Figure 5B. The purified enzyme lost its activity at pH values ranging from 3 to 7.5 and completely lost its activity at pH 3, as the enzyme activity was 40 times lower than that at pH 7.5 (*p* < 0.01). The activity obtained at pH 5.5 was 3.3 times lower than that at pH 7.5 (*p* < 0.05). The optimal pH was 7.5. (VIBVN)NAT retained most of its activity over a wide range of pH values (7.5 to 10) and began to lose activity after the pH value was higher than 8.5. The activity at pH 11.5 was 2.2 times lower than that at pH 7.5 (*p* < 0.05). Moreover, these results indicated that (VIBVN)NAT exhibited a preference of neutral and alkaline pH values, as the active-site cysteine needs to be deprotonated for activity.

### Kinetic Parameters Characterization

**FIGURE 6:**
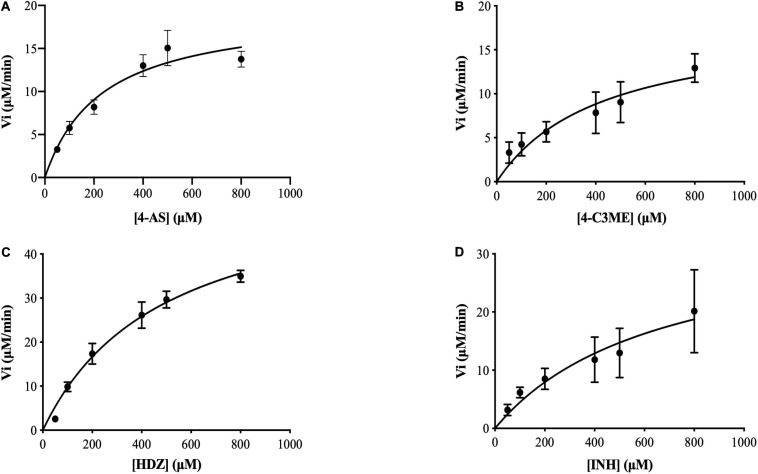
Determination of the *K*_*m*_ and *V*_*max*_ values of (VIBVN)NAT. The activity of NAT was detected by the DTNB method. Michaelis–Menten plots for the calculation of *K*_*m*_ and *V*_*max*_ for the enzyme with **(A)** 4-AS, **(B)** 4-C3ME, **(C)** HDZ, and **(D)** INH as the substrate. The data are presented as the mean ± SD of three independent experiments. Error bars indicate SD values.

To assay the kinetic parameters of (VIBVN)NAT, the enzyme activity against substrates, including HDZ, 4-AS, INH, and 4-C3ME, at various concentrations (50–800 μM) was analyzed. Figure 6 depicts the Michaelis–Menten kinetics plot, and the kinetic parameters measured for (VIBVN)NAT are summarized in Table 1. The *K*_*m*_ value reflects the binding affinity between the substrate and the enzyme. Our results showed that the *K*_*m*_ values of INH, HDZ, 4-C3ME, and 4-AS to (VIBVN)NAT were 978.8, 490.3, 425.7, and 232 μM, respectively. Although the binding activity of INH and HDZ to the enzyme was not high, the *k*_*cat*_ of the enzyme was higher than 1.0 s^–1^. A similar trend was observed in mycobacterial NATs (Sikora et al., 2008). These findings were consistent with previous research showing that HDZ has a high affinity for both (BACCR)NAT and (BACAN)NAT (Pluvinage et al., 2007; Kubiak et al., 2012). In this research, (VIBVN)NAT acetylated HDZ, 4-AS, INH, and 4-C3ME with different catalytic efficiencies (*k*_*cat*_/*K*_*m*_ ratios were 2.89, 2.073, 1.123, and 1.055 S^–1^mM^–1^, respectively, Table 1), indicating that (VIBVN)NAT has a high activity against several substrates.

**TABLE 1:** Kinetic parameters for *V. vulnificus* N-acetyltransferase determined from Michaelis–Menten kinetics plots.

**Substrate**	***K*_*m*_ (μM)**	***V*_*max*_ (μM min^–1^)**	***k*_*cat*_ (S^–1^)**	***k*_*cat*_/*K*_*m*_ (S^–1^ mM^–1^)**
4-AS	232.5	19.56	0.482	2.073
4-C3ME	425.7	18.22	0.449	1.055
HDZ	490.3	57.46	1.417	2.89
INH	978.8	44.56	1.099	1.123

### *T*_*m*_ and *T*_*agg*_ Characterization of *V. vulnificus* Arylamine *N*-acetyltransferase

**FIGURE 7:**
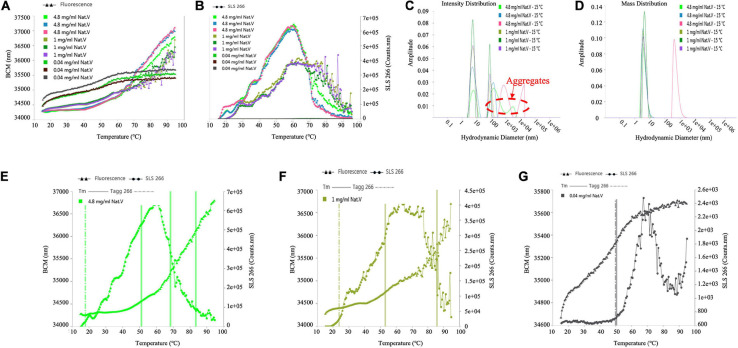
Determination of the *T*_*m*_, *T*_*agg*_ and size distribution of (VIBVN)NAT. **(A)** Depicts *T*_*m*_ value by denaturation curve. **(B)** Depicts *T*_*agg*_ value by aggregation curve. **(C)** Intensity Distribution. **(D)** Mass Distribution. **(E–G)** Depicts the relationship between denaturation and aggregation at different concentrations.

The *T*_*agg*_ values of different sample concentrations calculated from SLS signals were 17.7, 23.7, and 49.2°C, respectively, as shown in Table 2, for (VIBVN)NAT. Figures 7A,B indicates that the enzyme showed poor colloidal stability at a concentration of 4.8 mg/mL; although 1 mg/mL protein aggregated slightly later (23.7°C), it was still easily aggregated. *T*_*m*_ values calculated by the Barycentric mean (BCM) method were detected at approximately 52°C for all three enzyme concentrations. For the 4.8 mg/mL samples, *T*_*m*_ 2 and *T*_*m*_ 3 were found at 69.3 and 81.7°C, respectively. For 1 mg/mL samples, *T*_*m*_ 2 was detected at 84°C. For the 0.04 mg/mL samples, only a *T*_*m*_ of approximately 52°C was detected (Table 2), which meant that the thermodynamic stability of (VIBVN)NAT gradually declined with decreasing concentration. The lower the BCM value is, the more stable the protein structure; otherwise, the protein structure is disordered. From the BCM overlay curve, it can be seen that the structure of the 0.04 mg/mL sample is more flexible than other concentrations due to the low concentration. The Figures 7E–G depicted the relationship between denaturation and aggregation at 4.8, 1, and 0.04 mg/ml sample concentration, respectively. The results shown in Figure 7G revealed that 0.04 mg/mL protein aggregation occurred almost simultaneously at the *T*_*m*_, indicating that protein denaturation induced aggregation. Aggregates existed in both the 4.8 and 1 mg/mL samples (Figure 7C), which was in great agreement with the SLS results. The particle size components at approximately 6 nm accounted for the main mass (Figure 7D). The measurement of the particle size information of the 0.04 mg/mL sample using the DLS signal is limited due to the low sample concentration.

**TABLE 2:** *T*_*m*_ and *T*_*agg*_ values determined for *V. vulnificus* N-acetyltransferase (*T*_*m*_, the midpoint of the unfolding event; *T*_*m*_ 1, *T*_*m*_ 2, and *T*_*m*_ 3, the first, second, and third transitions, respectively; *T*_*agg*_, the starting point of the aggregation event; *T*_*agg*_ 266, static light scattering (SLS) signals recorded at 266 nm).

**Concentration range (mg/ml)**	**Average *T*_*m*_ 1 (°C)**	**% CV *T*_*m*_ 1**	**Average *T*_*m*_ 2 (°C)**	**% CV *T*_*m*_ 2**	**Average *T*_*m*_ 3 (°C)**	**% CV *T*_*m*_ 3**	**Average *T*_*agg*_ 266 (°C)**	**% CV *T*_*agg*_ 266**
4.8	51.3	1.15	69.3	1.2	81.7	1.86	17.7	2.82
1	53.5	0.93	84	5.54			23.7	1.69
0.04	52.3	3.06					49.2	1.83

### Molecular Modeling

**FIGURE 8:**
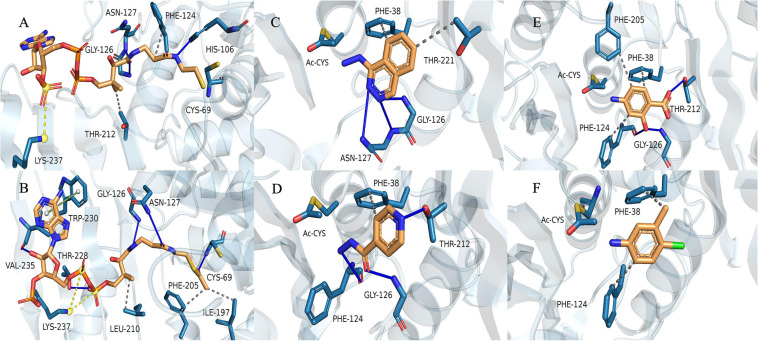
Binding model of CoA with (VIBVN)NAT. **(A)** The binding model of AcCoA with (VIBVN)NAT **(B)** and the binding model of (VIBVN)NAT with four aromatic amine substrates: **(C)** HDZ, **(D)** INH, **(E)** 4-AS, and **(F)** 4-C3ME. The solid blue lines represent hydrogen bonds, the gray dotted lines represent hydrophobic interactions, the yellow dotted lines represent salt bridges, and the green dotted lines represents pi-pi interactions.

We used the (MYCMR)NAT1 structure (PDB ID: 2VFC), which also lacks the “mammalian/eukaryotic insertion loop,” in complex with CoA as the reference. Homology models of (VIBVN)NAT generated using SWISS-MODEL revealed a Global Model Quality Estimation (GMQE) score, Qualitative Model Energy ANalysis (QMEAN) score, and sequence identity of 0.78, 0.31, and 42.86%, respectively. The binding mode shows that residues in the active pocket of (VIBVN)NAT can form hydrogen bonds (His106, Gly126, and Asn127), hydrophobic interactions (Phe124 and Thr212), and salt bridges (Lys237) with CoA (Figure 8A; Salentin et al., 2015; Schrödinger^2^). In the binding mode of (VIBVN)NAT with AcCoA, the long chain of β-mercaptoethylamine of AcCoA deeply inserts into the cavity containing a cysteine, which is similar to the binding mode of (VIBVN)NAT with CoA (Figure 8B). Several hydrophobic interactions were found between the carbon atom at the end of the acetyl group and amino acid residues (Ile197 and Phe205) in the active pocket of (VIBRIO)NAT. The hydrogen bond formed by a carbonyl oxygen atom of the acetyl group as Cys69 helps stabilize the intermediate transition state when the acetyl group transfers to cysteine from AcCoA (Fullam et al., 2008). The effects of chemical structures on enzyme substrate specificity have been well studied (Smith et al., 2003; Amin et al., 2013). As the most affinitive substrate, HDZ interacts with the enzyme through several interactions, such as hydrophobic interactions (Phe38 and Thr221) and hydrogen bonds (Gly126, Asn127) (Figure 8C). The residues of the active pocket form hydrophobic interactions (Phe38) and hydrogen bonds (Gly126, Phe124, and Thr212) with INH (Figure 8D). For 4-C3ME, some hydrophobic interactions were formed between the substrate and residues (Phe38 and Phe124) of the active pocket (Figure 8F). Hydrophobic interactions (Phe38, Phe124, and Phe205) and hydrogen bonds (Gly126 and Thr212) were formed between 4-AS and the active site of the enzyme (Figure 8E). The acetyl group transfer from AcCoA to the substrate is well known as the ping-pong mechanism.

## Discussion

*Vibrio vulnificus* is a human foodborne pathogen with striking public health importance. The potential pathogenicity of *V. vulnificus* in humans has been studied worldwide (Raghunath et al., 2008; Huehn et al., 2014; Suffredini et al., 2014; Caburlotto et al., 2016). Recent studies have indicated that bacteria using NAT as the key factor against environmental attack (Boukouvala and Fakis, 2005; Sim et al., 2008a). Therefore, knowledge of NAT properties may lead to a better understanding of pathogen virulence and provide a basis for further studies. The structure of the NAT enzyme in *V. vulnificus* can be used to decipher the NAT enzyme catalytic mechanism by understanding the basis of the substrate specificity and substrate binding mode. To date, all NATs have been described to share three typical distinct structural domains: the α-helical bundle (N-terminus), the central β-barrel, and the α/β lid (C-terminus) (Sinclair et al., 2000). Normally, the active site residues are in domains I and II, which are much more conserved than domain III. The C-terminal region of the eukaryotic NAT enzyme has an extension region that extends deeply to the active site and participates in its formation. The C-terminus of bacterial NATs forms a helix that is remote from the active site (Payton et al., 2001). Previous reports have shown that the length of the C-terminal region can control enzymatic activity and has a role in substrate specificity (Sim et al., 2008a). In the current study, the results of the (VIBVN)NAT sequence alignment showed that the C-terminal region of (VIBVN)NAT is approximately 20 amino acids shorter than the C-terminal region of other kinds of NATs (Figure 1). The activity of (VIBVN)NAT was tested by the PNPA and DTNB methods with different acetyl donors and acceptors. Interestingly, major differences in the activity profiles of the various substrates were observed when using the PNPA and DTNB assays. (VIBVN)NAT is much more active using the DTNB method than the PNPA method (Figure 2). The reason for this is not yet fully understood, but the loss of PNPA activity may be partly due to the lack of the sequence length of the C-terminus compared to those of the other eukaryotic and prokaryotic NATs (Mushtaq et al., 2002). AcCoA generally binds to NATs more tightly than PNPA (Andres et al., 1983; Mushtaq et al., 2002; Sandy et al., 2005b). This could explain why (VIBVN)NAT has various activity profiles using different acetyl donors. In terms of substrate specificity studies, the sensitivity of NATs from different species to aromatic amine substrates varies widely (Brooke et al., 2003; Westwood and Sim, 2007; Wu et al., 2007). In previous studies, NATs from prokaryotes and eukaryotes exhibited high affinity for 2-AF and 5-AS (Delomenie et al., 2001; Sandy et al., 2005a; Martins et al., 2008). HDZ and INH were used as classic aromatic hydrazine substrates of multiple NATs. The antitubercular drug INH is inactivated in the human body through acetylation by the NAT enzyme (Mitchell et al., 1975; Ellard and Gammon, 1976). (VIBVN)NAT catalyzed the AcCoA-dependent acetylation of several aromatic amines, including arylamine antibiotics (INH), but had little influence on SMX, which is similar to previous studies showing that other mycobacterial NAT enzymes acetylate and inactivate INH, thus improving the survivability of mycobacteria (Upton et al., 2001; Fullam et al., 2008; Sikora et al., 2008). Similar to (MYCA9)NAT, hydrogen bonds between residues around the catalytic cysteine of the enzyme and hydroxyl groups with 4-AS were observed, and these bonds are beneficial for improving the ability to transfer acetyl groups for 4-AS (Cocaign et al., 2014; Xu et al., 2015). The catalytic efficiency is similar to the values observed in other bacterial NATs (Sikora et al., 2008; Kubiak et al., 2012). Other than the C-terminal length, the difference between the eukaryotic and prokaryotic NAT structures is the existence of a 17-residue extension in eukaryotic NATs named the “mammalian-like insertion” or “eukaryotic interdomain loop”, which is a loop between domains II and III (Kubiak et al., 2013). This insertion has been reported to be absent in prokaryotic NAT structures, but recent studies have revealed that the insertion of 14-amino acids in the bacterial (BACAN)NAT1 structure is equivalent to the “mammalian insertion” in domain II (Wu et al., 2007; Grant, 2008). The presence of the “mammalian/eukaryotic insertion loop” will constrain the conformation of CoA binding with the protein, which impacts the binding mode of CoA by shaping a narrower cleft around the active cysteine and causes the geometry of CoA to bend in the section of pyrophosphate. Otherwise, the geometry of CoA is extended and fitted into an extended active cavity (Pluvinage et al., 2011; Xu et al., 2015). A previous study indicated that the “mammalian/eukaryotic insertion loop” occupied the 3′ADP-phosphate of CoA when binding with (MYCMR)NAT1 and therefore bending the CoA geometry (Kubiak et al., 2013). The role of this loop is not yet fully understood. It has been reported to contribute to the structural integrity of the protein by interacting with other residues in the protein (Walraven et al., 2007). Moreover, the presence of a “mammalian/eukaryotic insertion loop” in (HUMAN)NAT2 and (BACAN)NAT1 but not in (MYCMR)NAT1 (one human and two bacterial isoforms) suggests that this insertion contributes to the binding mode of the AcCoA cofactor, and the mode is different in various species. Although the “mammalian-like” insertion is not observable in our (VIBVN)NAT structure, it existed in both (BACCR)NAT and (BACAN)NAT, which are closely related to (VIBVN)NAT (Figure 1).

Enzyme stability is crucial for protein engineers. After studying the structural features of (VIBVN)NAT, we explored the chemical stability of (VIBVN)NAT under different conditions. Redox regulation is a mechanism that may affect xenobiotic biotransformation (Pagano, 2002). A previous study showed that H_2_O_2_ could oxidize the catalytic cysteine to sulfonic acid (-SOH) and therefore inactivate (HUMAN)NAT1 (Atmane et al., 2003). In this research, under treatment with 25 μM H_2_O_2_, the activity of (VIBVN)NAT was slightly impacted (preserving 95% of the enzyme activity) (Figure 4B), while in (HUMAN)NAT1, the enzyme activity was significantly inactivated (IC50 = 45 μM), which indicated that (VIBVN)NAT might have a specific antioxidizing effect. Redox regulation studies on (HUMAN)NAT1 also showed that the inhibition by H_2_O_2_ could be fully reversed by DTT and GSH. This result is consistent with our results that the H_2_O_2_-dependent inactivation of (VIBVN)NAT can be reversed by DTT (Figure 4C). It was also found that H_2_O_2_ has a similar regulatory mechanism to other catalytic cysteine-dependent enzymes (Lee et al., 2002; Vieceli Dalla Sega et al., 2017). The presence of metal ions also plays an important role in the biological function of many enzymes. The binding of metal ions with enzymes can cause a shift in active site residue coordinates, affecting the catalytic activity or structural stability of the enzyme (Sheen et al., 2012). In this study, the effects of several metal ions on the activity of (VIBVN)NAT were tested. Mn^2+^ was found to enhance the activity, Mg^2+^, K^+^, Na^+^, and Ca^2+^ did not affect the activity of (VIBVN)NAT, while Cu^2+^ and Zn^2+^ were found to significantly reduce the enzyme activity (*p* < 0.01) (Figure 4D). According to the theory of the hard/soft-acid/base (HSAB) principle, since Cu^2+^ and Zn^2+^ are borderline acids, they are more likely to combine with the sulfhydryl group of the catalytic cysteine and cause the enzyme to inactivate than other tested metal ions (Ouyang et al., 2018). This inactivation caused by CuSO_4_ could be fully reversed by EDTA (1 mM final concentration); however, ZnSO_4_ was found to irreversibly inhibit (VIBVN)NAT (Figure 4E). Furthermore, the role of temperature and pH on the chemical stability of (VIBVN)NAT was also assessed. The optimal growth environment of *V. vulnificus* is warm seawater, where water temperature ranges from 9 to 31°C (Heng et al., 2017). Interestingly, in our study, (VIBVN)NAT exhibited a slightly lower enzyme activity in cold buffer (4°C) than in room temperature buffer, suggesting that this enzyme still retains part of its function in a cold environment (Figure 5A).

In conclusion, the expression of NAT in Vibrio has important implications. This study is the first to report the functional and structural characterization of an antibiotic-modifying NAT enzyme from *V. vulnificus*. This provides a basis to understand the substrate-binding specificity of (VIBVN)NAT. Epidemiological investigations of *V. vulnificus* show that along with the overuse of antibiotics, the antibiotic drug resistance of this lethal bacterium is becoming an increasing concern. Due to the important role of NAT in bacterial growth and metabolism (Sim et al., 2012, 2014), (VIBVN)NAT could be essential to study the arylamine antibiotic resistance mechanism in *V. vulnificus*.

## Data Availability Statement

The raw data supporting the conclusions of this article will be made available by the authors, without undue reservation.

## Author Contributions

XL performed the experiments, analyzed the data, and drafted the manuscript. YL and GZ organized the data and drafted the figures. YZ, LL, JW, YW, SZ, XL, and DG commented the study and revised the manuscript. XX and PW designed, supervised the study, and revised the manuscript. All authors read and approved the final manuscript.

## Conflict of Interest

The authors declare that the research was conducted in the absence of any commercial or financial relationships that could be construed as a potential conflict of interest.

## Footnotes

1 http://espript.ibcp.fr/ESPript/cgi-bin/ESPript.cgi

2 https://pymol.org/2/support.html
